# Effect of a Nutrient-Rich, Food-Based Supplement Given to Rural Vietnamese Mothers Prior to or during Pregnancy on the Trajectories of Nutrient Biomarkers

**DOI:** 10.3390/nu12102913

**Published:** 2020-09-23

**Authors:** Janina Goletzke, Hoang T. Nga, Phi N. Quyen, Tu Ngu, Janet C. King

**Affiliations:** 1 Department of Nutritional Sciences and Toxicology, University of California Berkeley, Berkeley, CA 94720, USA; j.goletzke@fastmail.com; 2 National Institute of Nutrition, Hanoi 100000, Vietnam; vietnga1124@gmail.com (H.T.N.); quyenk37@gmail.com (P.N.Q.); tungu.nin@gmail.com (T.N.)

**Keywords:** food-based supplement, micronutrient intake, pregnancy, biomarker trajectories, rural Vietnamese women

## Abstract

Nutrient interventions initiated after conception tend to have modest effects on maternal nutritional status and pregnancy outcomes. Thus, we compared the association between micronutrient intakes and the trajectories of their biomarkers before and during pregnancy. Data from a randomized trial of the effect of a nutrient-rich, food-based supplement given to 317 Vietnamese women prior to or during pregnancy on birth outcomes were used to assess nutrient intakes with biomarker trajectories of zinc, iron, folate, cobalamin, and vitamin A using linear mixed regression models. The circulating plasma or serum trajectories of all five micronutrients were associated to their baseline levels (*p* < 0.0001). Plasma zinc trajectories were also related to farm work (*p* = 0.024). Cobalamin and vitamin A trajectories were associated with gestational weight gain (*p* = 0.003 and *p* = −0.037, respectively). In this population of rural Vietnamese women, nutrient intakes during pregnancy did not affect biomarker trajectories. The primary determinant of each nutrient biomarker trajectory was its respective baseline level prior to conception.

## 1. Introduction

It is well accepted that nutrition before and during pregnancy affects maternal and offspring health [1,2]. In addition to inadequate intakes of energy and protein i.e. due to limited food availability, the diets of pregnant women in lower- and middle-income countries (LMICs) are often insufficient in several micronutrients [3,4] due to low intakes of animal products or the poor micronutrient absorption due to the presence of phytate [5]. These micronutrient deficiencies could have detrimental health consequences for both the mothers and their infants [6].

Since adequate micronutrient intakes are critical for fetal development and because the micronutrient requirements increase by a larger percentage than energy [7,8,9], supplementation is often recommended for women of reproductive age [10]. Intervention studies of maternal micronutrient supplementation, typically initiated during the second trimester, providing either iron and folate, multi-micronutrients with or without lipids, or protein–energy supplements, have frequently had positive, but modest, effects on offspring anthropometrics such as birthweight [11,12,13]. Of note, in the 2019 update of the Cochrane review on supplementation during pregnancy, multi-micronutrient supplementation in comparison to iron (and folate) supplementation alone showed benefits on several birth outcomes [12]. Studies evaluating the association between nutrient intakes and biomarker status are needed to better understand the link between maternal nutrition and pregnancy outcomes. To date, studies relating maternal micronutrient intakes to birth outcomes have been inconsistent [14,15,16,17]. In addition, research has started to focus on the optimal time point of nutritional interventions, suggesting benefits of starting prior to conception [18,19]. Hence, comparisons of pre-pregnancy and gestational intakes to measurements of micronutrient status during pregnancy are needed.

We previously [20] reported that a food-based supplement initiated prior to conception or at mid-pregnancy in a low-resource Vietnamese population improved fetal growth-related birth outcomes but not infant growth during the first 24 months. In the present analysis, we evaluated the effects of initiating a micronutrient supplementation prior to pregnancy or at mid-gestation (16 weeks) on maternal nutritional status. The food-based supplement was designed to provide at least 50% of the pregnancy requirement for five micronutrients: folate, cobalamin, Vitamin A, iron, and zinc. First, we assessed the biomarker trajectories across pregnancy. Then, we determined whether the micronutrient intake was associated with circulating biomarker trajectories throughout pregnancy. Finally, we assessed the effect of other potential factors influencing biomarker trajectories throughout pregnancy such as farm-work status or gestational weight gain.

## 2. Materials and Methods

### 2.1. Study Design and Population

The present study is a secondary data analysis of the VINAVAC study (VINA stands for Vietnam, VAC stands for the VAC system: V for *Vuon* or garden, A for *Ao* or pond, and C for *Chuong* or cattle shed) that was conducted between 2011 and 2015 in Vietnam. It is described in detail elsewhere [21] and a flow diagram presenting the study participant progress is published in our previous publication [20]. Briefly, non-pregnant women who registered to marry were recruited for the study. At baseline, a medical doctor conducted an evaluation of maternal socio-demographic and anthropometric characteristics at the commune health center. A blood sample was collected, and anthropometric measurements were done at baseline as well. Additionally, dietary intake was assessed from 24-hr recalls, which were repeated twice on non-consecutive days. These dietary, anthropometric, and blood assessments were repeated at mid-gestation and late gestation (16 ± 3 and 32 ± 3 weeks, respectively).

After informed consent was obtained, the women were assigned randomly to one of the three interventions: (1) consumption of a food-based supplement 5 days/week from study recruitment (pre-pregnancy) to term (approximately 11 months) (named PC-T); (2) consumption of the food-based supplement 5 days/week from 16 weeks of gestation to term (approximately 5 months, named MG-T), or (3) routine prenatal care with no interventional supplementation (named RPC). Preparation of the supplement meals was done daily according to a 10-day cycle of menus. The basis of the menus was both local animal-source foods (including either pork, shrimp, liver, blood, or embryonated duck eggs) and dark-green leafy vegetables. The aim of the menus was to provide at least 50% of the recommended dietary allowance for pregnant women for the following five nutrients: iron, zinc, folate, vitamin A, and vitamin B12. Meal provision was done by health workers mid-morning 5 days/week at a designated eating site. These health workers also observed meal consumption and recorded the quantity consumed. For the present analyses, data of the 317 women finishing the study were used. 

Review and approval of the study was done by the Ethical and Scientific Committees of the National Institute of Nutrition, Hanoi, Vietnam, and by the Institutional Review Board of the Children’s Hospital Oakland Research Institute (Registered Clinical Trials.gov: NCT01235767). Informed consents were obtained at study enrollment prior to conception from each of the woman. 

### 2.2. Maternal Measurements

Dietary intakes were assessed from two repeated non-consecutive 24-h recalls at three time points: baseline, mid-pregnancy, and late pregnancy. Trained staff recorded the participants’ recall data in their home. To quantify food consumption, food scales, a set of commonly used utensils such as spoons, bowls, and cups, pictures of food, and common mixed food recipes were used. Coding of the dietary recalls was done using Vietnamese food composition tables [22]. Daily intakes of energy and macronutrients as well as vitamins and minerals were calculated. Women choosing to take multiple micronutrients or iron/folate supplements during pregnancy were not excluded. In our cohort, 23% took iron and folate supplements at the 16 weeks study visit and 39% took supplements at 32 weeks according to the 24-h recalls. In the present study, micronutrient intake includes the intervention food supplement, but it does not include the additional iron/folate supplements taken by some of the women in order to differentiate micronutrient intake from foods, which was the focus of our study, rather than that taken through capsules.

Anthropometric measurements (woman’s height, weight, mid-upper arm circumference, triceps and subscapular skinfold thickness, done in duplicate) were completed at baseline (pre-pregnancy), mid-pregnancy (16 weeks), and late pregnancy (32 weeks) by study staff. Additionally, a morning fasting blood sample was collected at the communal health station. The participants were contacted by the Communal Health Station Director twice a month to assess their menstruation status and conduct a morbidity interview.

In a laboratory at the field site, a separate EDTA-preserved whole blood tube was collected for hemoglobin measurements by the cyanmethemoglobin method to identify anemia (non-pregnant women: hemoglobin concentration < 12 g/dL; pregnant women: <11 g/dL) [20]. The remaining whole blood was centrifuged, and plasma and serum samples were stored at −20 °C. Within two weeks, they were transferred from the field laboratory to Hanoi for storage at −80 °C. Iron and zinc were measured at Children’s Hospital Oakland Research Institute by inductively coupled plasma optical emission spectrometry (ICP-OES) [23]. Serum folate and vitamin B12 (cobalamin) were analyzed by microbiological assay: a chloramphenicol-resistant strain of Lactobacillus casei and a colistin sulfate-resistant strain of Lactobacillus leichmannii was used for folate and B12, respectively [24,25]. The liquid chromatography-mass spectrometry (LC-MS) method was used to measure serum vitamin A. Additionally, two acute phase proteins were measured using ELISA kits (Immunology Consultants Laboratories, Portland, OR: plasma C-reactive protein (CRP) and serum α-1-acid glycoprotein (AGP)).

### 2.3. Statistical Analyses

The study population characteristics for continuous variables are presented as means and standard deviations or medians (25th, 75th percentile) for normally and not normally distributed continuous variables, respectively. Total numbers (n) and percentages were presented for categorical variables. As the living conditions, physical work demands, and food access differed between farm-working women and those with other occupations, participant characteristics are presented separately for farmworkers and non-farmworkers. They were compared using ANOVA for normally distributed continuous variables, the Kruskal–Wallis test for not normally distributed continuous variables, and the Chi-square test for categorical variables.

To assess longitudinal trajectories for serum folate, cobalamin, and vitamin A and for plasma zinc and iron, the mean or median values (for normally and not normally distributed variables, respectively), were presented by the intervention group as well as farm-worker status and analyzed using repeated measurements ANOVA for differences between study visits as well as ANOVA and Kruskal–Wallis tests for differences between intervention groups.

To examine the association between the respective dietary micronutrient intake and biomarker trajectories over the course of pregnancy, linear mixed-effect regression models (PROC MIXED in Statistical Analysis System (SAS)), including both random and fixed effects, were computed. To account for non-linear biomarker changes during the study duration, we included intercept and time as random effects. Non-normally distributed biomarkers were log-transformed to achieve normal distribution. Since associations between dietary micronutrient intake and biomarker trajectories did not differ by the dietary intervention group (*p*-interaction > 0.2), data from all three intervention groups were pooled for the subsequent analyses. Model “a” included the following fixed effects: time (defined as duration in weeks between the baseline and final visit), micronutrient intake at baseline, and the change in micronutrient intake defined as the difference in intakes between baseline and 32 weeks gestation. Thus, the analysis yielded 2 regression coefficients for the association between dietary intake and biomarker levels representing (1) the cross-sectional estimate (an estimate for the regression of baseline dietary micronutrient on the respective baseline biomarker level, e.g., association between dietary zinc intake and serum zinc levels at baseline) and, (2) the concurrent estimate (an estimate for the regression of the change in dietary micronutrient intake between the baseline and late pregnancy visit on the concurrent change in biomarker levels). In addition, the following relevant confounding variables, according to our own data and the literature, were simultaneously considered in the models: fixed effects of maternal baseline energy intake, age, body mass index (BMI) at baseline, gestational weight gain, work as a farmer, type of household latrine, baseline level of C-reactive protein, and group of randomization (e.g., preconception, mid-gestation, or routine care) (model a). Additionally, in a second model (model b), the models were adjusted for the baseline levels of the respective biomarker. 

All analyses were carried out by using SAS software (version 9.1.3; SAS Institute, Cary, NC, USA) and were performed with a significance level at *p* < 0.05.

## 3. Results

The characteristics of the whole study population are shown in Table 1. On average, the women were 21 years of age, and approximately half completed education through middle school. The majority of the women (78%) worked as farmers and lived with their parents-in-law (71%), which is typical in rural Vietnam. The mean/median baseline intakes of the five micronutrients under investigation were below the recommended intake for premenopausal women [26]. However, the mean measures of iron status (hemoglobin, hematocrit, ferritin, and plasma iron) were above deficiency cut-offs [27]. A total of 21.4% of the women were anemic at baseline. Mean plasma zinc levels were below and mean serum levels of folate, cobalamin, and vitamin A were above the suggested deficiency cut-offs [28,29,30,31]. Furthermore, CRP levels were low in our cohort.

Table 2 compares the characteristics of the women who worked as farmworkers with those who did not. The farmworkers were younger (*p* < 0001), less likely to be educated beyond middle school (*p* < 0.0001), primarily lived with their parents-in-law (*p* = 0.002), and had a covered household latrine (*p* < 0.0001). In addition, farmworking women tended to have a greater arm muscle area (*p* = 0.07) and had significantly lower gestational weight gains (*p* < 0.0001). Nutrient intake comparisons showed that there was no difference in energy intakes between the two groups (*p* = 0.7), but the farmworkers consumed a greater proportion of their energy as carbohydrates and consequently less calories from fat and protein compared to non-farmworkers (*p* = 0.003, 0.02, and 0.007 for carbohydrate, fat, and protein comparisons, respectively). No micronutrient intake differences were noted except that the farmworking women tended to have lower zinc intakes (*p* = 0.09). Nutritional status biomarkers did not differ except for that the farmworkers tended to have lower hemoglobin levels than non-farmworkers (*p* = 0.08).

**Figure 1:**
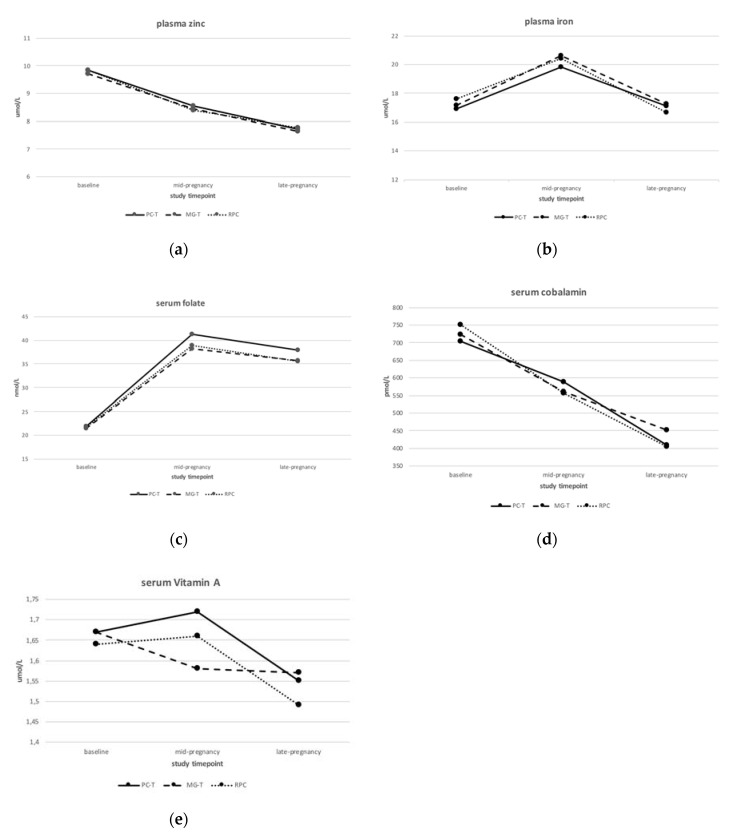
Biomarker trajectories from baseline visit (before conception) to late pregnancy by intervention group for (**a**) plasma zinc; (**b**) plasma iron; (**c**) serum folate; (**d**) serum cobalamin; (**e**) serum vitamin A. PC-T, consumption of a food-based supplement 5 days/week from study recruitment (pre-pregnancy) to term (approximately 11 months); MG-T, consumption of the food-based supplement 5 days/week from 16 weeks of gestation to term (approximately 5 months); RPC, routine prenatal care with no interventional supplementation.

Figure 1 presents biomarker trajectories from baseline to mid-pregnancy and to the late-pregnancy study visit for the five nutrients of interest in the three intervention groups (PC-T, MG-T, and RPC). None of the five nutritional biomarkers differed between the three intervention groups (PC-T, MG-T and RPC, *p* < 0.3) at any of the three time points. Plasma zinc and serum cobalamin levels significantly decreased from pre-pregnancy to 32 weeks gestation (*p* < 0.0001) in all three intervention groups. For plasma iron, a significant increase between the baseline and the mid-pregnancy visit was observed (*p* < 0.0001 in all three intervention groups), which was followed by a significant decrease close to baseline levels at 32 weeks (*p* < 0.0001). Serum folate levels also increased between baseline and mid-pregnancy (*p* < 0.0001), which were followed by a small but significant decrease in all three intervention groups from 16 to 32 weeks of gestation (*p* < 0.0001 in all groups). Serum vitamin A levels increased between baseline and mid-pregnancy for the PC-T and RPC groups (*p* < 0.0001), which was followed by a significant decline to less than baseline values by late pregnancy (*p* < 0.0001). For the MG-T group, serum vitamin A levels decreased until 16 weeks and then remained stable when their supplement was initiated at 16 weeks (Figure 1).

**Figure 2:**
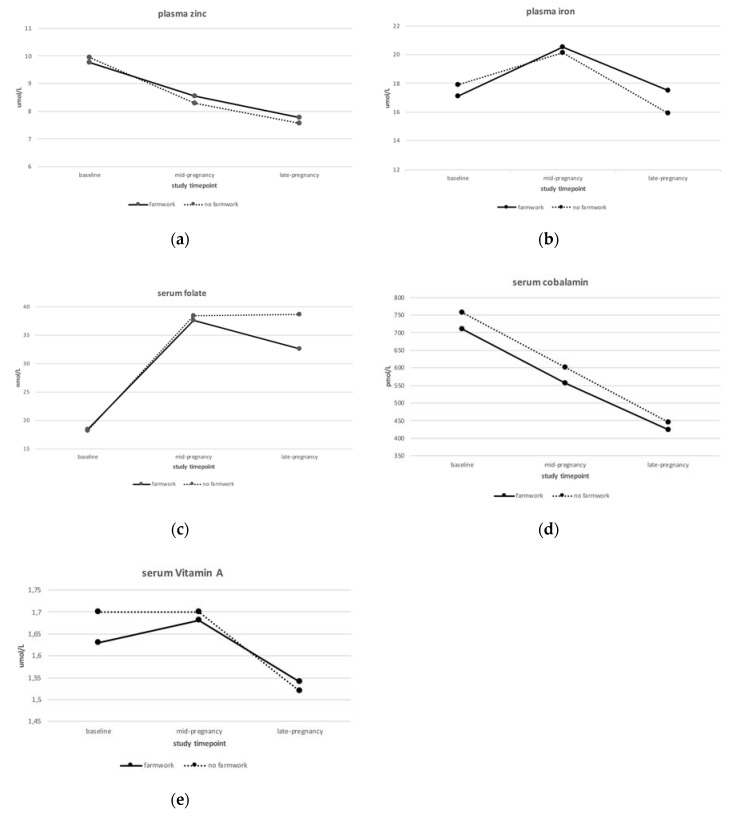
Biomarker trajectories from baseline visit (before conception) to late pregnancy by farm-work status for (**a**) plasma zinc; (**b**) plasma iron; (**c**) serum folate; (**d**) serum cobalamin; and (**e**) serum vitamin A.

The biomarker trajectories for those women doing farm work versus those who did not are shown in Figure 2. These trajectories are similar with those in Figure 1 showing the changes due to treatment group. Thus, no differences in the biomarker trajectories according to farm-work status were observed (*p* ≥ 0.2).

The results of the linear mixed effects models using data from the entire study sample are shown in Table 3. For each nutrient biomarker, models were run separately to examine the relevance of the respective micronutrient intake as well as other covariates (simultaneously combined in one model) on the trajectories from the baseline (before conception) to the late pregnancy (gestational week 32) study visit. Regarding plasma zinc trajectories, energy intake at baseline was negatively associated with the plasma zinc trajectory (*p* = 0.045, model 1a). After including baseline zinc levels in the model, this association was no longer significant (*p* = 0.3, model 1b). However, being a farmworker was significantly associated with plasma zinc levels (*p* = 0.024). In comparison to those women working as farmers, those who did not were more likely to have decreasing zinc trajectories. In addition, baseline zinc status was significantly positively associated with zinc trajectories (*p* < 0.0001, model 1b), indicating that those women with higher baseline zinc levels were more likely to have an increase in plasma zinc levels throughout pregnancy. For plasma iron, no significant associations were observed in model a. The inclusion of baseline iron status in model 2b showed that it was positively associated with iron trajectories (*p* < 0.0001). Regarding serum folate trajectories, a statistically significant positive association was observed with baseline CRP levels (*p* = 0.043, model 3a). After inclusion of baseline folate levels, only a trend for this association remained (*p* = 0.099, model 3b). In addition, baseline serum levels of folate were independently and positively associated with folate trajectories across pregnancy (*p* < 0.0001, model 3b). For serum cobalamin levels, a trend for a negative association was observed with baseline energy intake (*p* = 0.057, model 4a). After the inclusion of baseline cobalamin levels, a significant negative association was observed with gestational weight gain: A higher gestational weight gain was longitudinally related to decreasing serum cobalamin levels (*p* = 0.003). Moreover, baseline cobalamin levels were independently and positively associated with cobalamin trajectories (*p* < 0.0001, model 4b). Lastly, for serum vitamin A, a positive longitudinal association between higher gestational weight gain and increasing serum vitamin A levels was observed (*p* = 0.004, model 5a), which persisted after the inclusion of baseline vitamin A levels (*p* = 0.037, model 5b). In addition, baseline vitamin A levels were positively related to vitamin A trajectories (*p* < 0.0001, model 5b).

As complete data of biomarker levels at all three study visits was not present for all participating women, we ran sensitivity analyses including only those with data on both the baseline and the late pregnancy visit. Those analyses did not differ from those run in the whole cohort (see Table S1).

## 4. Discussion

In this study, the effect of a food-based intervention initiated prior to conception or at mid-gestation on maternal nutrient biomarkers was assessed in rural Vietnamese women. The food-based intervention was designed to provide at least 50% of the recommended nutrient intake for five nutrients: zinc, iron, folate, vitamin B12, and vitamin A. The food-based supplement had no effect on biomarker blood levels nor did the overall micronutrient intake of the women. The only consistent and independent determinant of each nutrient biomarker trajectory was its respective baseline level measured before pregnancy.

Although many studies have examined the effect of maternal supplementation during pregnancy on birth outcomes, data from both human and animal studies suggests an advantage of improving maternal nutritional status prior to conception on fetal growth and postnatal health [32]. However, only two groups evaluated the impact of preconception supplementation in low-resource populations [33,34]. In the Women First Trial [34,35], a multicountry study done in the DR Congo, Guatemala, India, and Pakistan, fetal growth outcomes were improved if the supplement was provided before conception or late in the first trimester; the effect on maternal nutritional status has not been reported. In another Vietnam study, either a weekly multiple micronutrient supplement or a weekly iron/folate supplement were given to the women for at least 3 months prior to conception [33]. Women receiving either supplement prior to conception had higher ferritin levels at three months postpartum and their infants had greater iron stores. However, the preconception supplement did not influence the incidence of anemia during pregnancy. As presented in an earlier publication by our group, the food-based supplement given to the women in this study also failed to alter infant anthropometric measurements at birth, which was the primary endpoint of our study [20]. The low weight gains, possibly due to the demanding farm work throughout the reproductive cycle, may have obviated any effects of the low energy, nutrient-rich food supplement on birth outcomes. However, by including data on baseline biomarker status, the present results confirm the importance of baseline (before conception) nutritional status, since that was the strongest predictor of subsequent blood nutrient trajectories during pregnancy. In our study, the preconception supplementation was initiated at study enrollment, and women were discontinued from the study if they did not conceive within 1 year. The average supplement duration in the PC-T group was 11 months total (preconception to 32-weeks), showing that most women conceived within 3 months after starting supplementation. A study conducted in Mumbai had a similar outcome. That study showed that a nutritional supplement given prior to conception until term only had an increasing effect on birth weight if the mother was not underweight and had received the supplement for at least 3 months prior to conception [18].

The effect of maternal micronutrient supplementation on nutritional biomarker status has been studied in several LMICs [16,17,36] and a Dutch study [14]. For example, in a Bangladeshi intervention study [16], supplementation with multiple micronutrients at the beginning of pregnancy improved late pregnancy micronutrient status (32 weeks), but micronutrient deficiencies persisted. Similar to our results, the authors found that the baseline status was strongly associated with late-pregnancy status, and they concluded that preconception supplementation may be warranted to meet nutritional demands in undernourished populations. In a Dutch study [14], changes in micronutrient intake and status between pre-conception and 24 weeks of gestation were determined in a cohort where women at risk of developing gestational diabetes mellitus were oversampled. Measures of micronutrient status were associated with supplemental intakes for some, but not all micronutrients. For example, plasma folate levels showed a pattern similar to our results (increasing until 12 weeks and then decreasing to 24 weeks of gestation), and the changes were related to supplemental but not dietary folate intake. Serum B12 levels again showed the same decreasing pattern we observed in our study, but neither dietary nor supplemental B12 intake were related to serum B12 levels. Plasma iron levels were not examined, but serum ferritin levels were not associated with dietary or supplemental iron intake. [14] Another Bangladesh trial examined the combined effect of food and micronutrient supplementation on biomarker concentrations at week 30 of pregnancy [17]. Women in the multiple micronutrient regimen had higher concentrations of plasma vitamin B12 at gestational week 30, but no other differences were observed between food and micronutrient regimens. Although starting the micronutrient supplement early (9 weeks) did not differ from initiating it at 20 weeks of gestation, a dose–response relationship between the number of capsules taken and plasma folate and ferritin concentrations was detected [17]. An intervention study in rural Nepal on the association between antenatal micronutrient supplementation and biochemical status indicators found a reduction in folate deficiency from the first to the third trimester (32 weeks), but there was only a modest effect on B12 and no effect on zinc status [36]. Compared to our study sample, these women had lower baseline biomarker levels. The variable changes in plasma volume expansion during pregnancy and potential inconsistencies in taking the prenatal supplements likely contribute to the insignificant effects of the supplement on serum or plasma levels.

In contrast to the intervention studies presented above [16,17,36], where the supplements provided at least 1 recommended dietary allowance (RDA) for pregnant women for each nutrient included, the food-based supplement in our study provided only half of the iron and zinc RDA, but it more than doubled the vitamin A, B12, and folate intakes [20]. The rationale for choosing to increase micronutrient intake with a food-based supplement and not pills were local preferences; i.e., at the time of this study, Vietnamese women were not normally given micronutrient supplements. The lower levels of iron and zinc may indeed explain the lack of an effect on the circulating levels of those nutrients. The dietary intake analyses for our study indicated a successful intervention with consistent or increasing micronutrient intakes from preconception to mid-gestation or from mid-gestation to late gestation, respectively, according to intervention group [20]. For folate and ferritin, dose–response associations have been shown between intake and concentrations of circulating biomarker levels [17,37]. Hence, higher dosage might have led to detectable associations in our study. In addition, differences between food-based supplements and supplementation regimens using capsules must be acknowledged. The nutrient absorption may be higher from capsules particularly if taken during fasting. However, food-based supplements are cost-effective alternatives with the potential to create long-lasting changes in maternal dietary habits. A recent study modeling various scenarios shows that food-supplements based on local foods could meet the micronutrient needs of women of reproductive age [38]. In addition, Ziaei et al. [17] did not observe any differences in nutrient biomarker levels when food-based supplements were compared to micronutrient tablets.

When examining biomarker status and trajectories during pregnancy, it is important to keep in mind that plasma volume increases during pregnancy [39,40]. This increase might be lower in low-income countries due to lower gestational weight gain, implicating a potentially bigger impact on biomarker changes. However, it is difficult to assess whether differences in plasma volume expansion affected our outcomes. Hematocrit levels might be a proxy measure of plasma volume changes. Thus, we included hematocrit levels in additional statistical analyses, but the results did not change (data not shown). Another possible explanation for the lack of response in biomarkers might be endocrine regulations occurring in pregnancy that facilitate nutrient transfer to the fetus without altering maternal status. Finally, both negative and positive micronutrient interactions might occur when micronutrients are provided simultaneously [36].

To the best of our knowledge, this is the first study showing that a food-based supplement providing modest amounts of key nutrients and only a small amount of energy did not influence the trajectory of those nutrients during gestation. The main strengths of our study are the repeatedly collected biomarker concentrations before and during pregnancy. In addition, the availability of data on several potential confounders, i.e., the socioeconomic situation of the women, further strengthens our analysis. For example, the comparisons of biomarker trajectories between women conducting farm work and those who did not are unique. A limitation of this study is the comparably small sample size that prevented conducting more stratified data analyses. In addition, only 68% of the women who consented completed the study, reducing our statistical power. Furthermore, it has to be kept in mind that the study was not designed to answer the present research question, as this is a post hoc data analysis. However, the precisely and repeatedly collected data allowed for these additional analyses to be done appropriately.

All together, the lack of an effect of dietary intake (both overall and from the food supplement) on biomarker levels in our study was unexpected and deserves further clarification in future studies. Different approaches to improve maternal nutrition need to be assessed, and both the timing of interventions as well as specific adaptations for different populations need to be examined. As discussed above, the lack of an effect in our study might have several reasons ranging from the type of intervention to specific aspects of the study population. However, while we do not dismiss the importance of an adequate dietary intake before and during pregnancy and the need to improve the maternal nutritional status prior to conception, the present results also emphasize the need to assess specific physiological circumstances of pregnancy, i.e., plasma volume increase, particularly in a LMIC setting.

## 5. Conclusions

In conclusion, our results indicate that nutrient intakes prior to and during pregnancy did not influence nutrient biomarker trajectories throughout gestation. The most consistent and independent determinant of the biomarker trajectory for each nutrient was its respective baseline level before pregnancy, highlighting the importance of improving maternal nutritional status prior to conception.

## Figures and Tables

**Table 1:** Characteristics of the study population at baseline (*n* = 317) *.

	Mean (SD)/Median (25th, 75th Percentile)
Age at Random Assignment, Year	21.4 (2.85)
Highest educational level, %	
elementary school	2.3%
middle school	56.6%
high school	20.6%
occupational school or higher	20.6%
Living arrangement, %	
with parents-in-law	71.4%
with husband only	10.5%
with parents	18.1%
Household latrine, %	
non/field/bush	1.0%
uncovered	5.1%
covered	63.8%
flush	30.1%
Work as farmers, %	77.9%
Anthropometry	
weight, kg	45,9 (4.85)
height, cm	152.8 (5.09)
MUAC, cm	24.02 (1.80)
AMA, cm^2^	23.3 (4.83)
AFA, cm^2^	16.4 (4.38)
BMI, kg/m^2^	19.7 (1.77)
Nutrient intakes ^1^	
energy intake, kcal/day	1748 (355)
carbohydrate intake, en%	65.5 (6.90)
fat intake, en%	18.8 (6.14)
protein intake, en%	16.0 (2.01)
iron intake, mg/day	12.7 (3.69)
zinc intake, mg/day	9.14 (2.26)
vitamin A intake, μg/day	476 (284, 681)
folate intake, μg/day	314 (144)
vitamin B12 intake, μg/day	1.78 (0.79, 2.71)
Nutritional status measurements ^2^	
hemoglobin, g/dL	12.9 (1.20)
hematocrit, %	40.3 (2.93)
ferritin, μg/L	43.3 (25.71, 83.34)
plasma iron, μmol/L	17.2 (6.61)
plasma zinc, μmol/L	9.79 (1.41)
serum vitamin A, μmol/L	1.64 (0.40)
serum folate, nmol/L	21.7 (10.7)
serum cobalamin, pmol/L	723 (572, 895)
CRP, mg/L	0.20 (0.10, 0.50)

* Values are means (SD) for normally distributed and median (25th, 75th percentile) for not normally distributed variables. For categorical variables, percentage of the total is presented. ^1^ The FAO/WHO recommended nutrient intakes for premenopausal women: iron: 29.4 mg/d (assuming 10% bioavailability); zinc: 9.8 mg/d (assuming low bioavailability); vitamin A: 500 μg RE/d; folate: 400 μg DFE/d; vitamin B12: 2.4 μg/d [26]. ^2^ deficiency cut-offs: hemoglobin <110 g/L, ferritin: <15 ug/L [27]; zinc: <10.7 mol/L [29]; vitamin A: <0.70 mol/L [30]; folate: <6.8 nmol/L, cobalamin: <150 pmol/L [28,31]. AFA, arm fat area; AMA, arm muscle area; BMI, body mass index; MUAC, middle upper arm circumference; CRP, C-reactive protein.

**Table 2:** Characteristics of the study population at baseline presented separately for farmworkers and non-farmworkers (*n* = 317).^1^

	Farmworkers *n* = 247 (77.9%)	Non-Farmworkers *n* = 70 (22.1%)	*p*^2^ for Difference
age at random assignment, year	21.0 (2.84)	22.7 (2.54)	<0.0001
Highest educational level, %			<0.0001
elementary school	2.9%	0%	
middle school	68.2%	14.7%	
high school	19.8%	23.5%	
occupational school or higher	9.1%	61.8%	
Living arrangement, %			0.002
with parents-in-law	75.5%	56.5%	
with husband only	10.6%	10.1%	
with parents	13.9%	33.3%	
Household latrine, %			<0.0001
non/field/bush	1.2%	0%	
uncovered	5.8%	2.9%	
covered	69.6%	42.7%	
flush	23.5%	54.4%	
Anthropometry			
weight, kg	46.0 (4.80)	45.6 (4.94)	0.3
height, cm	153 (5.07)	153 (5.16)	0.3
MUAC, cm	24.1 (1.77)	23.8 (1.90)	0.3
AMA, cm^2^	23.5 (4.69)	22.6 (5.29)	0.07
AFA, cm^2^	16.3 (4.38)	16.4 (4.44)	0.7
BMI, kg/m^2^	19.7 (1.72)	19.4 (1.92)	0.1
gestational weight gain, kg	6.67 (2.96)	9.65 (4.28)	<0.0001
Nutrient intakes ^3^			
energy intake, kcal/day	1751 (375)	1739 (286)	0.7
carbohydrate intake, en%	66.1 (6.99)	63.5 (6.33)	0.003
fat intake, en%	18.4 (6.22)	20.3 (5.71)	0.02
protein intake, en%	15.8 (1.93)	16.6 (2.18)	0.007
iron intake, mg/day	12.5 (3.21)	13.3 (5.00)	0.3
zinc intake, mg/day	9.06 (2.33)	9.4 (2.02)	0.09
vitamin A intake, μg/day	476 (291, 677)	462 (264, 793)	0.6
folate intake, μg/day	315 (146)	309 (138)	0.9
vitamin B12 intake, μg/day	1.75 (0.75, 2.66)	1.98 (0.83, 3.26)	0.1
Nutritional status measurements ^4^			
hemoglobin, g/dL	12.8 (1.21)	13.1 (1.14)	0.08
hematocrit, %	40.3 (2.94)	40.1 (2.95)	0.5
ferritin, μg/L	44.2 (25.1, 88.6)	42.8 (26.4, 78.2)	0.7
plasma iron, μmol/L	17.1 (6.13)	17.9 (8.59)	0.9
plasma zinc, μmol/L	9.8 (1.42)	10.0 (1.34)	0.4
serum vitamin A, μmol/L	1.6 (0.40)	1.70 (0.39)	0.2
serum folate, nmol/L	21.4 (10.7)	22.3 (11.0)	0.6
serum cobalamin, pmol/L	711 (574, 895)	757 (575, 896)	0.5
CRP, mg/L	0.20 (0.10, 0.60)	0.20 (0.10, 0.50)	0.8

^1^ Values are means (SD) for normally distributed and median (25th, 75th percentile) for not normally distributed variables. For categorical variables, percentage of the total is presented. ^2^ ANOVA for normally distributed continuous variables, Kruskal–Wallis test for not normally distributed continuous variables, and Chi-square test for categorical variables. ^3^ The FAO/WHO recommended nutrient intakes for premenopausal women: iron: 58 mg/d (assuming 5% bioavailability); zinc: 9.8 mg/d (assuming low bioavailability); vitamin A: 500 g RE/d; folate: 400 g DFE/d; vitamin B12: 2.4 g/d [26]. ^4^ deficiency cut-offs: hemoglobin <110 g/L, ferritin: <15 ug/L [27]; zinc: <10.7 mol/L [29]; vitamin A: <0.70 mol/L [30]; folate: <6.8 nmol/L, cobalamin: <150 pmol/L [28,31]. AFA, arm fat area; AMA, arm muscle area; MUAC, middle upper arm circumference; CRP, C-reactive protein.

**Table 3:** Mixed models of biomarker trajectories over the course of pregnancy (*n* = 317).

	**1.** **Plasma Zinc Trajectories**			
	**(a)** **Mixed Model**		**(b)** **Mixed Model Including Baseline Plasma Zinc**	
	**ß (SE)**	***p*-Value**	**ß (SE)**	***p*-Value**
baseline dietary zinc intake, mg/day	0.0813 (0.0601)	0.2	0.0385 (0.0407)	0.3
concurrent change in dietary zinc intake, mg/day	−0.0057 (0.0254)	0.8	−0.0059 (0.0171)	0.7
baseline energy intake, kcal/day	−0.0007 (0.0003)	0.045	−0.0003 (0.0002)	0.3
intervention group	0.0264 (0.2034)	0.9	0.1321 (0.1403)	0.3
maternal age, years	0.0262 (0.0283)	0.4	0.0081 (0.0193)	0.7
maternal BMI before pregnancy, kg/m^2^	0.0076 (0.0460)	0.9	−0.0305 (0.0320)	0.3
farm work (Ref group: farm work)	−0.2745 (0.1909)	0.2	−0.2962 (0.1294)	0.02
household latrine (no vs. flush latrine, flush = Ref)	−0.7587 (0.6840)	0.3	0.0465 (0.4595)	0.9
gestational weight gain, kg	−0.0266 (0.0254)	0.3	−0.0236 (0.0171)	0.2
baseline CRP, mg/L	−0.0438 (0.0620)	0.5	0.0374 (0.0562)	0.5
baseline plasma zinc levels, mmol/L			0.5135 (0.0375)	<0.0001
	**2.** **Plasma Iron Trajectories**			
	**(a)** **Mixed Model**		**(b)** **Mixed Model Including Baseline Plasma Iron**	
baseline dietary iron intake, mg/day	−0.0775 (0.1088)	0.5	−0.0125 (0.0796)	0.9
concurrent change in dietary iron intake, mg/day	0.0221 (0.0535)	0.7	0.0103 (0.0396)	0.8
baseline energy intake, kcal/day	0.0009 (0.0012)	0.5	0.0004 (0.0009)	0.7
intervention group	−0.8230 (1.0093)	0.4	0.8653 (0.7723)	0.3
maternal age, years	0.0660 (0.1351)	0.6	0.0918 (0.1004)	0.4
maternal BMI before pregnancy, kg/m^2^	−0.0725 (0.2211)	0.7	−0.1478 (0.1661)	0.4
farm work (Ref group: farm work)	−0.3270 (0.9178)	0.7	−0.7109 (0.6788)	0.3
household latrine (no vs. flush latrine, flush = Ref)	−2.1156 (3.2385)	0.5	−1.1767 (2.3558)	0.5
gestational weight gain, kg	0.0137 (0.1201)	0.9	0.0572 (0.0884)	0.5
baseline CRP, mg/L	−0.1726 (0.2983)	0.6	0.4588 (0.3002)	0.1
baseline plasma iron levels, mmol/L			0.5106 (0.0419)	<0.0001
	**3.** **Serum Folate Trajectories**			
	**(a)** **Mixed Model**		**(b)** **Mixed Model Including Baseline Serum Folate**	
	**ß (SE)**	***p*-Value**	**ß (SE)**	***p*-value**
baseline dietary folate intake, mg/day	−0.0003 (0.0003)	0.2	−0.0002 (0.0002)	0.3
concurrent change in dietary folate intake, mg/day	0.0001 (0.0002)	0.8	0.0001 (0.0001)	0.4
baseline energy intake, kcal/day	0.000004 (0.0001)	1	0.00002 (0.0001)	0.8
intervention group	0.0979 (0.0871)	0.3	0.0390 (0.0652)	0.6
maternal age, years	0.0213 (0.0116)	0.07	0.0091 (0.0087)	0.3
maternal BMI before pregnancy, kg/m^2^	0.0044 (0.0190)	0.8	0.0026 (0.0141)	0.9
farm work (Ref group: farm work)	0.0355 (0.0777)	0.6	0.0520 (0.0577)	0.4
household latrine (no vs. flush latrine, flush = Ref)	0.0799 (0.2829)	0.8	0.1731 (0.2071)	0.4
gestational weight gain, kg	−0.0074 (0.0102)	0.5	−0.0043 (0.0076)	0.6
baseline CRP, mg/L	0.0493 (0.0242)	0.04	0.0297 (0.0179)	0.099
baseline serum folate levels, nmol/L			0.5507 (0.0492)	<0.0001
	**4.** **Serum Cobalamin Trajectories**			
	**(a)** **Mixed Model**		**(b)** **Mixed Model Including Baseline Serum Cobalamin**	
baseline dietary cobalamin intake, mg/day	0.0157 (0.0148)	0.3	−0.0044 (0.0064)	0.5
concurrent change in dietary cobalamin intake, mg/day	0.0046 (0.0071)	0.5	−0.0016 (0.0031)	0.6
baseline energy intake, kcal/day	−0.0002 (0.0001)	0.06	0.00002 (0.00004)	0.6
intervention group	0.0344 (0.0762)	0.7	0.0009 (0.0337)	1
maternal age, years	0.0086 (0.0106)	0.4	−0.0014 (0.0046)	0.8
maternal BMI before pregnancy, kg/m^2^	−0.0194 (0.0176)	0.3	−0.0124 (0.0076)	0.1
farm work (Ref group: farm work)	−0.0129 (0.0718)	0.9	0.0116 (0.0311)	0.7
household latrine (no vs. flush latrine, flush = Ref)	0.3166 (0.2569)	0.2	0.1519 (0.1089)	0.2
gestational weight gain, kg	−0.0097 (0.0096)	0.3	−0.0125 (0.0041)	0.003
baseline CRP, mg/L	−0.0198 (0.0224)	0.4	−0.0067 (0.0096)	0.5
baseline serum cobalamin levels, pmol/L			0.7128 (0.0311)	<0.0001
	**5.** **Serum Vitamin A Trajectories**			
	**(a)** **Mixed Model**		**(b)** **Mixed Model Including Baseline Serum Vitamin A**	
baseline dietary vitamin A intake, mg/day	0.0001 (0.0001)	0.1	0.0001 (0.0001)	0.03
concurrent change in dietary vitamin A intake, mg/day	0.00003 (0.00003)	0.3	0.00003 (0.00002)	0.1
baseline energy intake, kcal/day	−0.0001 (0.0001)	0.2	−0.00003 (0.00004)	0.4
intervention group	−0.0194 (0.0666)	0.8	0.0102 (0.0440)	0.8
maternal age, years	−0.0063 (0.0086)	0.5	0.0011 (0.0057)	0.8
maternal BMI before pregnancy, kg/m^2^	0.0073 (0.0142)	0.6	−0.0071 (0.0094)	0.4
farm work (Ref group: farm work)	−0.0103 (0.0579)	0.9	−0.0411 (0.0380)	0.3
household latrine (no vs. flush latrine, flush = Ref)	0.0714 (0.2064)	0.7	0.1157 (0.1329)	0.4
gestational weight gain, kg	0.0224 (0.0077)	0.004	0.0107 (0.0051)	0.04
baseline CRP, mg/L	−0.0122 (0.0182)	0.5	0.0062 (0.0119)	0.6
baseline serum vitamin A levels, mmol/L			0.5641 (0.0395)	<0.0001

Models contain a random statement with an unstructured covariance structure. Model a contains time (defined as duration in weeks between the baseline and final visit), micronutrient intake at baseline, and the change in micronutrient intake that was defined as the difference in intakes between baseline and 32 weeks gestation; fixed effects of baseline energy intake, maternal age, maternal body mass index (BMI) at baseline, gestational weight gain, working as a farmer, household latrine, baseline levels of C-reactive protein, and group of randomization. Model b additionally contains baseline levels of the respective biomarker.

## Supplementary Materials

The following are available online at https://www.mdpi.com/2072-6643/12/10/2913/s1, Table S1: Multivariable prediction models of biomarker trajectories over the course of pregnancy: sensitivity analysis including only those women with at least data on the baseline and late pregnancy study visit.
