# Supplementary material for: Effect of a Nutrient-Rich, Food-Based Supplement Given to Rural Vietnamese Mothers Prior to or during Pregnancy on the Trajectories of Nutrient Biomarkers
Source: Nutrients. 2020 Sep 23;12(10):2913. doi: 10.3390/nu12102913 (PMC7598161; doi:10.3390/nu12102913)
Supplement: Supplementary file 1 [file nutrients-12-02913-s001.pdf]

Supplemental Table 1: Multivariable prediction models of biomarker trajectories over the course of pregnancy: sensitivity analysis including only those women with at least data on the baseline and late pregnancy study visit.

	1. plasma zinc trajectories (n=197)			
	(a) multivariable prediction model		(b) multivariable prediction model including baseline plasma zinc	
	ß (SE)	p-value	ß	p-value
baseline dietary micronutrient intake*	0.1124 (0.0608)	0.07	0.0487 (0.0409)	0.2
concurrent change in dietary micronutrient intake *	0.0005 (0.0254)	1.0	-0.0024 (0.0170)	0.9
baseline energy intake	-0.0009 (0.0004)	0.02	-0.0003 (0.0002)	0.2
intervention group	0.0057 (0.2088)	1.0	0.0866 (0.1401)	0.5
maternal age	0.0216 (0.0287)	0.5	0.0059 (0.0193)	0.8
maternal BMI before pregnancy	-0.0175 (0.0474)	0.7	-0.0310 (0.0318)	0.3
farmwork (Ref group: farmwork)	-0.3023 (0.1951)	0.1	-0.2910 (0.1301)	0.03
household latrine (group 0 vs 3, 3=Ref)	-0.8369 (0.6780)	0.2	0.0267 (0.4561)	1.0
gestational weight gain	-0.0260 (0.0254)	0.3	-0.0230 (0.0169)	0.2
baseline CRP	0.0020 (0.0825)	1.0	0.0337 (0.0556)	0.5
+ baseline plasma zinc levels			0.5075 (0.0378)	<0.0001

	2. plasma iron trajectories (n=198)			
	(a) multivariable prediction model		(b) multivariable prediction model including baseline plasma iron	
baseline dietary micronutrient intake*	-0.0620 (0.1097)	0.6	-0.0112 (0.0802)	0.9
concurrent change in dietary micronutrient intake *	0.0249 (0.0546)	0.6	0.0105 (0.0401)	0.8
baseline energy intake	0.0009 (0.0012)	0.5	0.0003 (0.0009)	0.7
intervention group	-0.8225 (1.0510)	0.4	0.8690 (0.7835)	0.3
maternal age	0.0892 (0.1386)	0.5	0.0935 (0.1016)	0.4
maternal BMI before pregnancy	-0.0139 (0.2281)	1.0	-0.1488 (0.1677)	0.4
farmwork (Ref group: farmwork)	-0.2256 (0.9462)	0.8	-0.7105 (0.6923)	0.3
household latrine (group 0 vs 3, 3=Ref)	-1.8659 (3.2527)	0.6	-1.7272 (2.3716)	0.5
gestational weight gain	0.0122 (0.1216)	0.9	0.0583 (0.0889)	0.5
baseline CRP	-0.5296 (0.3953)	0.2	0.4574 (0.3022)	0.1
+ baseline plasma iron levels			0.5093 (0.0423)	<0.0001

	3. serum folate trajectories (n=191)			
	(a) multivariable prediction model		(b) multivariable prediction model including baseline serum folate	
	ß (SE)	p-value	ß (SE)	p-value
baseline dietary micronutrient intake*	-0.0003 (0.0003)	0.2	-0.0002 (0.0002)	0.3
concurrent change in dietary micronutrient intake *	0.0001 (0.0002)	0.6	0.0001 (0.0001)	0.3
baseline energy intake	0.00004 (0.0001)	0.6	0.00003 (0.0001)	0.6
intervention group	0.0981 (0.0878)	0.3	0.0422 (0.0658)	0.5
maternal age	0.0175 (0.0117)	0.1	0.0081 (0.0088)	0.4
maternal BMI before pregnancy	0.0087 (0.0191)	0.6	0.0037 (0.0143)	0.8
farmwork (Ref group: farmwork)	0.0578 (0.0781)	0.5	0.0534 (0.0583)	0.4
household latrine (group 0 vs 3, 3=Ref)	0.0833 (0.2808)	0.8	0.1678 (0.2082)	0.4
gestational weight gain	-0.0087 (0.0102)	0.4	-0.0043 (0.0076)	0.5
baseline CRP	0.0501 (0.0240)	0.04	0.0307 (0.0179)	0.09
+ baseline serum folate levels			0.5405 (0.0498)	<0.0001

	4. serum cobalamin trajectories (n=190)			
	(a) multivariable prediction model		(b) multivariable prediction model including baseline serum cobalamin	
baseline dietary micronutrient intake*	0.0097 (0.0133)	0.5	-0.0029 (0.0064)	0.6
concurrent change in dietary micronutrient intake *	0.0009 (0.0065)	0.9	-0.0006 (0.0032)	0.8
baseline energy intake	-0.0001 (0.0001)	0.099	0.00001 (0.00004)	0.8
intervention group	0.0590 (0.0701)	0.4	0.0088 (0.0339)	0.8
maternal age	0.0047 (0.0097)	0.6	-0.0016 (0.0046)	0.7
maternal BMI before pregnancy	-0.0160 (0.0158)	0.3	-0.0124 (0.0076)	0.1
farmwork (Ref group: farmwork)	-0.0397 (0.0652)	0.5	0.0146 (0.0313)	0.6
household latrine (group 0 vs 3, 3=Ref)	0.3834 (0.2281)	0.095	0.1634 (0.1085)	0.1
gestational weight gain	-0.0055 (0.0086)	0.5	-0.0129 (0.0041)	0.002
baseline CRP	-0.0137 (0.0199)	0.5	-0.0060 (0.0095)	0.5
+ baseline serum cobalamin levels			0.6994 (0.0318)	<0.0001

	5. serum vitamin A trajectories (n=191)			
	(a) multivariable prediction model		(b) multivariable prediction model including baseline serum vitamin A	
baseline dietary micronutrient intake*	0.0001 (0.0001)	0.1	0.0001 (0.0001)	0.04
concurrent change in dietary micronutrient intake *	0.00003 (0.00003)	0.3	0.00003 (0.00002)	0.1
baseline energy intake	-0.0001 (0.0001)	0.3	-0.00003 (0.00004)	0.4
intervention group	-0.0113 (0.0687)	0.9	0.0082 (0.0446)	0.9
maternal age	-0.0048 (0.0089)	0.6	0.0014 (0.0057)	0.8
maternal BMI before pregnancy	0.0061 (0.0145)	0.7	-0.0076 (0.0095)	0.4
farmwork (Ref group: farmwork)	-0.0180 (0.0593)	0.8	-0.0427 (0.0385)	0.3
household latrine (group 0 vs 3, 3=Ref)	0.0836 (0.2088)	0.7	0.1161 (0.1339)	0.4
gestational weight gain	0.0233 (0.0078)	0.004	0.0107 (0.0051)	0.04
baseline CRP	-0.0116 (0.0184)	0.5	0.0066 (0.0120)	0.6
+ baseline serum vitamin A levels			0.5636 (0.0398)	<0.0001

Models contain a random statement with an unstructured covariance structure.
Model a contains time (defined as duration in weeks between the baseline and final visit), micronutrient intake at baseline and the change in micronutrient intake that defined as the difference in intakes between baseline and 32 weeks gestation; fixed effects of baseline energy intake, maternal age, maternal BMI at baseline, gestational weight gain, working as a farmer, household latrine, baseline levels of C-reactive protein, and group of randomization
Model b additionally contains baseline levels of the respective biomarker
